# Lipid droplet biogenesis in the ovary

**DOI:** 10.1002/rmb2.12618

**Published:** 2024-12-15

**Authors:** Megumi Ibayashi, Satoshi Tsukamoto

**Affiliations:** ^1^ Laboratory Animal and Bioresource Sciences Section National Institutes for Quantum Science and Technology Chiba Japan; ^2^ Kato Ladies Clinic Shinjuku‐ku Tokyo Japan

**Keywords:** angiogenesis, follicle, lipid droplet, mouse, ovary

## Abstract

**Background:**

Lipid droplets (LDs) are organelles consisting of a central core of neutral lipids covered by a single layer of phospholipids and are found in most eukaryotic cells. Accumulating evidence suggests that LDs not only store neutral lipids but also coordinate with other organelles for lipid metabolism within cells.

**Methods:**

This review focuses on the synthesis of LDs during follicular development and highlights the factors involved in the regulation of LD biogenesis within the ovary.

**Main Findings:**

In the mammalian ovary, the presence of LDs has long been recognized mainly by morphological analysis. However, their distribution in the ovary varies according to the region and cell type; for example, LDs are abundant in the medulla, which has a rich blood vessel network, in interstitial cells, which are the site of steroid production, and surrounding growing follicles, while they are poor in granulosa cells within follicles. LDs are also enriched in the corpus luteum after ovulation and massively accumulate in atretic follicles during follicular growth. Furthermore, LD synthesis is synchronized with angiogenesis during follicular development.

**Conclusion:**

Addressing the functional link between LD biogenesis and angiogenesis is essential for understanding the molecular basis underlying LD biology, as well as the ovarian dysfunction with metabolic disorders.

## Graphical abstract

Our review focuses on the synthesis of LDs during follicular development and highlights the factors involved in the regulation of LD biogenesis within the ovary.

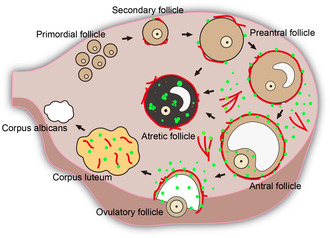

## 1 LIPID DROPLETS

Lipid droplets (LDs) are organelles that consist of a core of neutral lipids, such as triglycerides and cholesterol esters, covered by a phospholipid monolayer.^1^, ^2^ Depending on intracellular energy levels, LDs alter their interactions with other organelles.^3^ LDs are found in most eukaryotic cells, but their size and number vary among cell types and tissues. Moreover, even within the same cell type, the characteristics of LDs (e.g., phospholipid composition of the LD surface and LD‐binding proteins) are not identical.^4^

Many of the proteins involved in neutral lipid synthesis are localized in the endoplasmic reticulum (ER), and thus LDs are believed to originate from the ER.^5^, ^6^ The formation of LDs in the ER consists of the following steps: (1) accumulation of proteins and enzymes involved in neutral lipid synthesis; (2) synthesis and accumulation of neutral lipids in the intermembrane space; (3) formation of lens structures with increasing neutral lipid content; (4) budding of LDs from the ER; and (5) maturation of the dissociated LDs.^2^, ^7^, ^8^ These sites of LD formation in the ER are determined by seipin (a protein encoded by the Berardinelli‐Seip congenital lipodystrophy type 2 [BSCL2]),^9^, ^10^ and its partner, lipid droplet assembly factor 1 (LDAF1).^11^

Recent proteomic analyses have identified more than 150 LD‐associated proteins. A complete description of these proteins is available in the LD Knowledge Portal (https://lipiddroplet.org/), an open‐access resource.^12^ LD‐associated proteins are roughly classified into two groups: class I and class II.^13^ Class I proteins, such as associated with LD protein 1, (ALD1, also known as methyl transferase‐like 7B), and diacylglycerol O‐acyltransferase 2 (DGAT 2) and acyl‐CoA synthetase long‐chain family member 3 (ACSL3), are localized in the ER and translocate from the ER to the LD surface when LD synthesis occurs.^14^, ^15^, ^16^ In contrast, class II proteins, including CTP: phosphocholine cytidylyltransferase α isoform (CCTα), and perilipins (PLINs), are distributed in the cytoplasm and localize to the surface of mature LDs that are dissociated from the ER.^17^, ^18^, ^19^

Recent studies have shown that LDs interact with other organelles.^3^ For example, nutritional depletion (reduced cellular energy levels) contributes to the transfer of fatty acids from LDs to mitochondria (i.e., contributes to the activation of β‐oxidation) via interactions between LDs and mitochondria.^20^ Similarly, reduced energy levels stimulate the autophagic degradation of LDs (lipophagy) by facilitating interactions between LDs and lysosomes.^21^ These interactions between LDs and other organelles occur via membrane contact sites.^22^ For example, PLIN5, which is abundant in tissues with high mitochondrial content, such as cardiac muscle and brown adipose tissue,^23^, ^24^ is involved in the interactions of LDs and mitochondria.^25^

## 2 OVARIAN FOLLICULAR DEVELOPMENT

An ovarian follicle is composed of an oocyte and surrounding somatic cells such as granulosa cells (GCs) and theca cells. Follicular development requires bidirectional communication between the oocyte and somatic cells via various factors.^26^, ^27^ When a primordial follicle harboring an oocyte covered by a single layer of flattened GCs is activated, the GCs proliferate to become a cuboidal structure and these cuboidal GCs surround the oocyte (primary follicle). In a secondary follicle, multiple layers of GCs cover the oocyte. During further follicular development, an extracellular matrix, the zona pellucida, forms in the gap between the oocyte and GCs, while theca cells proliferate around the GCs. Follicular growth after the secondary follicle stage is gonadotropin‐dependent, and further development results in an antral follicle with a lumen filled with follicular fluid. At this stage, the GCs differentiate into mural GCs, which localize around the basement membrane and play an endocrine role, and cumulus cells, which encase and nourish the oocyte.

Subsequently, an increase in luteinizing hormone (LH surge) causes the rupture of a portion of the follicular wall, which releases the cumulus cell‐covered oocyte from the follicle (ovulation). After ovulation, the follicle becomes the corpus luteum, a temporary endocrine organ that produces progesterone, which is necessary to maintain pregnancy. However, if pregnancy is not established, the corpus luteum must regress to allow the initiation of a new reproductive cycle. Only a few follicles are able to generate mature oocytes, indicating that the majority of follicles regress as atretic follicles during follicular development.

## 3 VISUALIZATION OF LD BIOGENESIS IN THE OVARY

For the efficient production of the energy and hormones necessary for follicular development (as described above), it may be reasonable to store their precursors, such as triglycerides and cholesterol esters, within LDs. Indeed, the presence of LDs in the ovary has long been recognized.^28^, ^29^ Notably, the distribution of LDs in the ovary varies according to the region and cell type; for example, LDs are abundant in the medulla, which has a rich vascular network, in interstitial cells, which are rich in hormone‐producing cells, and around the developing follicles, while LDs are poor in intrafollicular GCs.

How are these differences in LD distribution regulated at the molecular level and what is its physiological significance? To answer these questions, it is important to determine when and how LDs are synthesized in the ovary. Conventional methods for observing LDs in organs and tissues have included immunostaining using antibodies against LD‐associated proteins and neutral lipid‐labeling reagents (e.g., Oil Red O and Nile Red) in addition to electron microscopic analysis. Although these methods can be used to investigate the distribution and ultrastructure of LDs within cells, it is difficult to visualize the entire process of LD biogenesis, including the formation of LDs on the ER, their subsequent growth, and intracellular distribution after budding from the ER.

Recently, in *Drosophila* and zebrafish, LD dynamics in vivo were investigated by expressing a fusion protein of PLINs and a fluorescent protein.^30^, ^31^, ^32^ However, because PLINs are cytoplasmic LD‐associated protein (class II protein, described above), only mature (neutral lipid‐rich) LDs can be labeled with this type of reporter protein. Therefore, LD formation that occurs in the ER (i.e., nascent LDs) cannot be visualized. Furthermore, LD‐associated proteins are involved in regulating LD size and number, and thus, the overexpression of such proteins in vivo is a potential concern for an excessive increase or accumulation of LDs within cells, resulting in metabolic dysfunction such as obesity.

**FIGURE 1:**
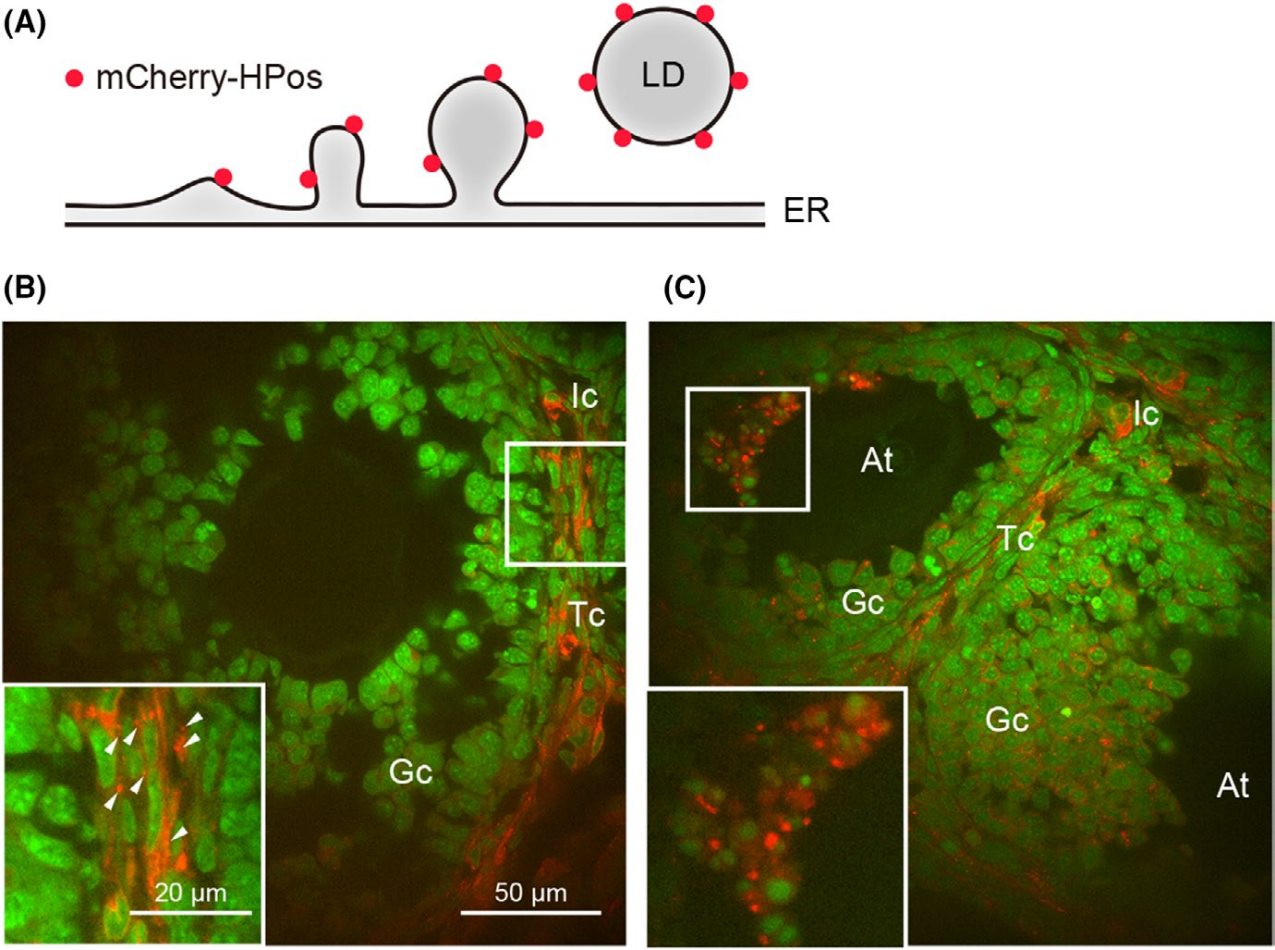
Visualization of mCherry‐HPos as an LD synthesis marker in the ovary. (A) Schematic illustration of mCherry‐HPos. mCherry‐HPos localizes to the surface of nascent to mature LDs synthesized at the ER, which allows visualization of LD biogenesis. (B, C) Confocal images of frozen ovarian sections from mCherry‐HPos mice illustrate that mCherry‐HPos foci (representing nascent LDs) are found in Tc and Ic (B, arrowheads). Note that atretic follicles contain numerous mCherry‐HPos foci of various sizes in GCs (C). Nuclei were counterstained with DRAQ5 (green) and merged images are shown at the bottom. At, atretic follicles; Gc, granulosa cells; Ic, interstitial cells; Tc, theca cells. Inset shows enlarged images of the boxed area.

Hydrophobic‐positive sequence (HPos) is a minimal sequence consisting of the C‐terminal sequence of caveolin 1 (20 amino acids) and the positively charged sequence of ALD1 (33 amino acids), which are required for insertion into the ER or translocation from the ER to LD surface, respectively.^14^, ^33^ Since HPos has the characteristics of class I proteins, a fusion protein of HPos and mCherry (red fluorescent protein; mCherry‐HPos) can be used to visualize the entire process of LD biogenesis (Figure 1A). Consistently, LiveDrop (encoding a 56‐amino acid, hydrophobic, membrane hairpin domain of glycerol‐3‐phosphate acyltransferase 4 [GPAT4]), which has similar properties to HPos, also allows the visualization of LD biogenesis.^5^, ^34^ These proteins are observed as small foci (representing nascent LDs) when LD synthesis is activated.

To visualize LD synthesis in vivo, we previously generated mCherry‐HPos mice that systemically express mCherry‐HPos.^35^ They are apparently healthy and fertile in both sexes. Fluorescence observation of frozen ovarian sections from mCherry‐HPos mice enables us to detect when and where LDs are actively synthesized during ovarian follicular growth.

Our studies using mCherry‐HPos mice revealed that low level of LD synthesis occurs in a defined region around the immature follicle (from the primary to secondary follicle stages).^35^ However, as follicular growth proceeds, LD synthesis is activated around theca cells and interstitial cells (Figure 1B). These cells are rich in steroid‐producing cells, which are positive for Ad4BP/SF‐1 (Nr5a1) and 3β‐hydroxysteroid dehydrogenase (3β‐HSD),^36^ suggesting that LD synthesis is induced with hormone production during follicular development. In GCs, in contrast, LD synthesis rarely occurs until ovulation. Notably, LD synthesis is activated temporarily by the LH surge (ovulation induced by human chorionic gonadotropin injection), but is subsequently suppressed and then reactivated after the ovulatory phase. During the ovulatory phase, LD synthesis is also detectable in GCs far from the theca layer.

Since only a small number of follicles develop completely, the majority of follicles degenerate (to become atretic follicles) during follicular development. In atretic follicles, mCherry‐HPos is observed as large structures in addition to numerous tiny foci (Figure 1C). This may be due to a reduction of the metabolic (lipolytic) activity of the synthesized LDs, leading to their accumulation, which is likely to be one of the mechanisms stimulating follicular atresia.

Although the molecular basis underlying the changes in LD synthesis levels before and after ovulation remains unclear, it is presumable that the luteinization of GCs may induce LD synthesis. Alternatively, during ovulation, LD synthesis also occurs in GCs far from the theca layer (around the oocyte), indicating that oocyte‐secreted factors suppress LD synthesis in the surrounding GCs until ovulation, but after ovulation, the effect of these factors is weakened and hence LD synthesis is induced. Consistently, the rapid induction of LD formation when GCs isolated from preovulatory follicles are cultured in vitro also supports the hypothesis that oocyte‐derived factors are involved in the regulation of LD synthesis. Given that oocytes secrete a wide variety of factors that influence the differentiation and function of the surrounding GCs and/or cumulus cells, it would be worthwhile investigating the mechanisms by which oocyte‐derived paracrine factors regulate LD synthesis. In this regard, oocyte‐derived transforming growth factor beta (TGFβ) superfamily proteins, including growth differentiation factor 9 (GDF9) and bone morphogenetic protein 15 (BMP15), as well as fibroblast growth factor 8 (FGF8), which collaborates with BMP15 to regulate of glycolysis in cumulus cells,^37^, ^38^ may also be involved.

Previous studies suggested that luteinization induces LD synthesis.^35^, ^39^ Since the corpus luteum functions as a temporary endocrine gland that produces progesterone, which is necessary for sustaining pregnancy, LD synthesis may induce the storage of the intracellular precursors of steroid hormones such as cholesterol esters. However, our recent study has revealed that, unexpectedly, LD synthesis is suppressed in part of the corpus luteum during pregnancy.^39^ Combined with the previous finding that LD depletion occurs in the functional corpus luteum (formed after pregnancy) in rodents,^40^ it seems likely that LDs synthesized after luteinization are degraded rapidly once pregnancy is established, and LD synthesis is suppressed until shortly before delivery. These unexpected findings also suggest the existence of an LD‐independent pathway for progesterone production in the functional corpus luteum during pregnancy.

## 4 REGULATION OF LD SYNTHESIS IN THE OVARY: POSSIBLE LINK BETWEEN LD SYNTHESIS AND ANGIOGENESIS

Angiogenesis, a process in which new blood vessels are formed from preexisting vessels,^41^ is closely associated with follicular development and corpus luteum formation.^42^ During follicular development, angiogenesis is induced in the periphery of the theca layer, and growing follicles are surrounded by an extensive blood vessel network.^43^ This vascular network contributes to the transport of nutrients, oxygen, and hormones necessary for follicular development, as well as for the removal of waste products (metabolites). However, since angiogenesis does not occur inside the follicle (i.e., GCs are avascular until ovulation), it is assumed that nutrients and oxygen are supplied to GCs from the thecal vessel via the basement membrane. In contrast, when the basement membrane of the follicle collapses due to ovulation, vascular endothelial cells invade the follicular lumen and form a new vascular network to generate the corpus luteum. The corpus luteum is thus enriched with a vascular network and becomes an endocrine gland for the secretion of progesterone,^44^, ^45^ which is necessary to maintain pregnancy.

**FIGURE 2:**
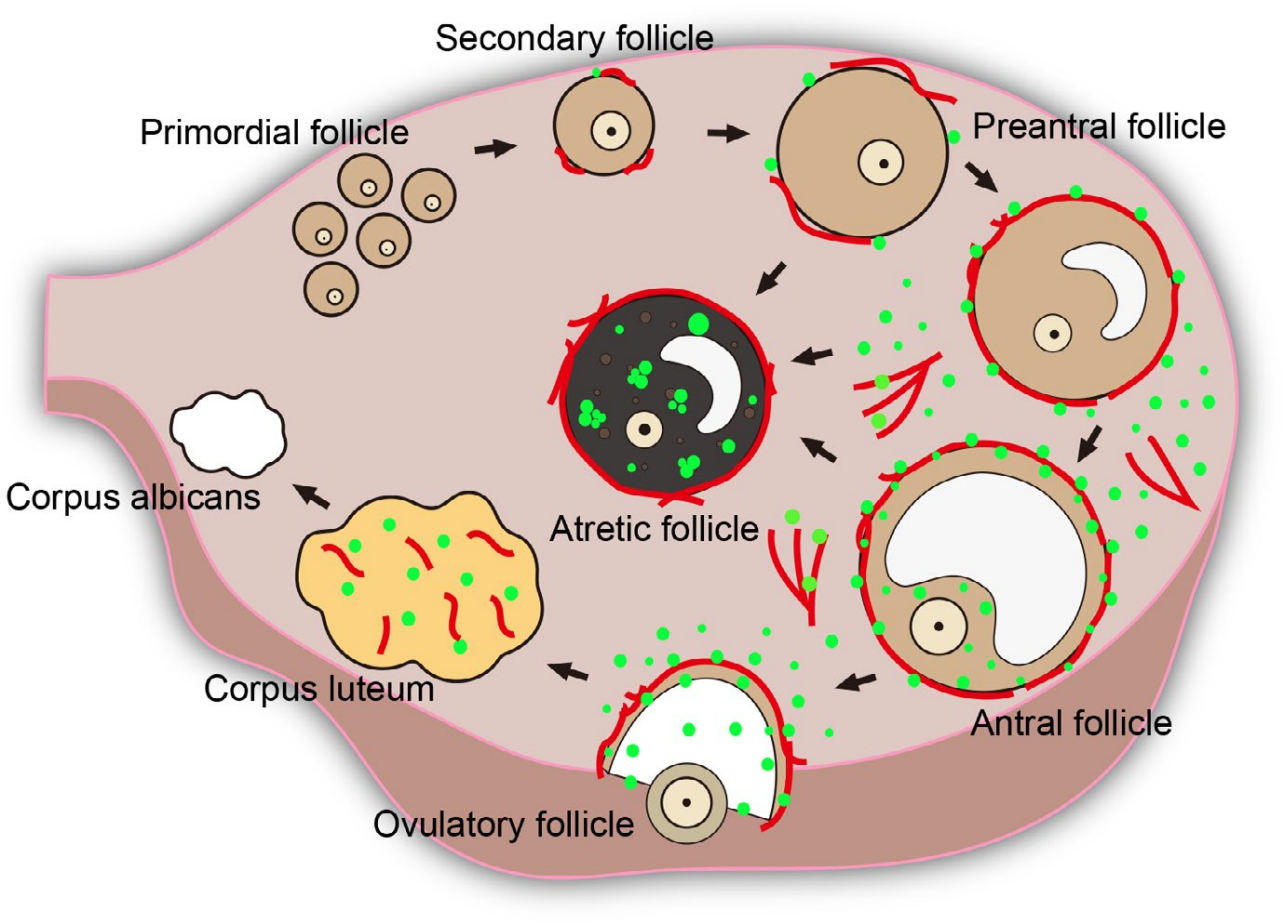
Close association between angiogenesis and LD synthesis in the ovary. Ovarian angiogenesis occurs from the early stages of follicle development and continues during follicular growth and luteinization. LD synthesis is enhanced in these regions of active angiogenesis. Red, blood vessels; green, LDs.

Intriguingly, in regions in which angiogenesis occurs during follicular development, LD synthesis is also induced,^35^ indicating a functional link between angiogenesis and LD biogenesis (see Figure 2). Supporting this hypothesis, intraovarian administration (bursa injection) of axitinib, a potent inhibitor of angiogenesis,^46^ influences LD biogenesis around developing follicles, which results in the accumulation of LDs.^35^ These findings indicate a close link between angiogenesis and LD biogenesis during follicular growth.

## 5 CONCLUSION

On the basis of recent studies focusing on LDs in the ovary, there is growing evidence that LD synthesis changes in a spatiotemporal manner during follicular development. Given the ovary is composed of a variety of cells necessary for oocyte production and pregnancy maintenance, the role of LDs may differ in a cell type‐dependent manner. In addition, recent studies have revealed that LDs are not simply lipid depots, but also play more roles than previously thought, in conjunction with other organelles. The functional link between LD synthesis and angiogenesis, which is activated during follicular development, requires further study. Since LD synthesis is induced during follicular development, especially in regions in which angiogenesis is active, it is also possible that blood‐derived factors, such as vascular endothelial growth factor, regulate LD synthesis. The finding that LD synthesis is suppressed in avascular GCs may also support this hypothesis. In summary, studies focusing on the regulation of LD synthesis during follicular development will contribute to our understanding of the molecular mechanisms involved in the creation of high‐quality oocytes and the ovarian dysfunction with metabolic disorders.

## CONFLICT OF INTEREST STATEMENT

The authors declare no conflict of interest.

## HUMAN RIGHTS STATEMENT AND INFORMED CONSENT

In this review, the author did not conduct any experiments using human‐derived materials.

## ANIMAL STUDIES

All mouse experiments were approved and registered by the Animal Care and Use Committee of the National Institutes for Quantum Science and Technology (approval number: 16‐1012‐5).
